# Digital media in intergenerational communication: Status quo and future scenarios for the grandparent–grandchild relationship

**DOI:** 10.1007/s10209-022-00957-w

**Published:** 2022-12-03

**Authors:** Nicola Döring, Veronika Mikhailova, Karlheinz Brandenburg, Wolfgang Broll, Horst-Michael Gross, Stephan Werner, Alexander Raake

**Affiliations:** 1 Media Psychology and Media Design Group, Technische Universität Ilmenau, Ilmenau, Germany; 2 Electronic Media Technology Group, Technische Universität Ilmenau, Ilmenau, Germany; 3 Virtual Worlds and Digital Games Group, Technische Universität Ilmenau, Ilmenau, Germany; 4 Neuroinformatics and Cognitive Robotics Lab, Technische Universität Ilmenau, Ilmenau, Germany; 5 Audiovisual Technology Group, Technische Universität Ilmenau, Ilmenau, Germany

**Keywords:** Grandparent–grandchild relationship, Older adults, Communication technologies, Digital media, Social robot, Augmented reality

## Abstract

Communication technologies play an important role in maintaining the grandparent–grandchild (GP–GC) relationship. Based on Media Richness Theory, this study investigates the frequency of use (RQ1) and perceived quality (RQ2) of established media as well as the potential use of selected innovative media (RQ3) in GP-GC relationships with a particular focus on digital media. A cross-sectional online survey and vignette experiment were conducted in February 2021 among *N* = 286 university students in Germany (mean age 23 years, 57% female) who reported on the direct and mediated communication with their grandparents. In addition to face-to-face interactions, non-digital and digital established media (such as telephone, texting, video conferencing) and innovative digital media, namely augmented reality (AR)-based and social robot-based communication technologies, were covered. Face-to-face and phone communication occurred most frequently in GP-GC relationships: 85% of participants reported them taking place at least a few times per year (RQ1). Non-digital established media were associated with higher perceived communication quality than digital established media (RQ2). Innovative digital media received less favorable quality evaluations than established media. Participants expressed doubts regarding the technology competence of their grandparents, but still met innovative media with high expectations regarding improved communication quality (RQ3). Richer media, such as video conferencing or AR, do not automatically lead to better perceived communication quality, while leaner media, such as letters or text messages, can provide rich communication experiences. More research is needed to fully understand and systematically improve the utility, usability, and joy of use of different digital communication technologies employed in GP–GC relationships.

## Introduction 

The world population is aging at a fast pace: It is expected that by 2050 one in six people in the world will be over the age of 65 [1]. As a result, grandchildren more and more often have the opportunity to develop long-lasting relationships with their grandparents which comes with benefits for the wellbeing of both sides [2]. Regular and rich communication plays an important role in maintaining a positive grandparent–grandchild (GP–GC) relationship [3]. However, factors such as geographical distance, limited mobility, or pandemic-related contact restrictions decrease face-to-face communication frequency, eventually putting older adults at risk of social isolation and loneliness [4, 5]. Communication technologies can help to overcome these problems by ensuring close contact over distance, fostering intergenerational connections, and ultimately strengthening GP–GC relationships [6–9].

Investigating mediated forms of contact between grandchildren and grandparents becomes especially relevant in the current age of the Internet. Previous research has repeatedly shown that older adults are less likely to use digital media and technological devices compared to younger generations [10]. However, since the beginning of the COVID-19 pandemic, more and more older adults have started using the Internet to maintain social contacts [10]. Recent findings show that 81% of senior citizens in Germany regularly go online [11], as opposed to only about 58% in the pre-pandemic times [12]. Internet-based forms of communication, such as video conferencing, have become particularly widespread among the older generation [13]. At the same time, innovative technologies, particularly from the fields of augmented reality (AR) and social robotics, are also being increasingly developed and studied for the target group of senior citizens to support their communication needs [14–18].

Against this background, the current study investigates the frequency of use and perceived quality of established non-digital and digital media as well as the potential use and perceived quality of innovative digital media (AR-based and social robot-based technologies) in GP–GC relationships.

## Related work

### Grandparent–grandchild relationships

Relationships between grandparents and grandchildren can constitute life-long social bonds that are equally important for both grandparents and grandchildren [2]. For grandparents, a positive relationship with their grandchildren is a source of enjoyment and pride [19]. It brings a sense of belonging and helps reduce social isolation and loneliness which increase at older age due to decreasing health and widowhood [4]. For the younger generation, having a meaningful relationship with their grandparents shapes their attitude toward older adults [20], has a positive effect on the ability to cope with life challenges [21, 22], and contributes to better self-esteem and mental health later in life [23].

GP–GC relationships tend to change in the course of life [24]. During adolescence and young adulthood, many grandchildren move away from their parents’ home and start spending more time away from family, thus, contacts with grandparents become less frequent and emotional closeness decreases [3, 25]. At the same time, during this transition the GP–GC relationship matures and becomes less dependent on other family members: Young adults become more likely to initiate contact with their grandparents and enjoy the intergenerational relationship [22, 26].

### Communication in grandparent–grandchild relationships

Intergenerational communication, defined as communication between members of older and younger generations, can occur in different forms [27]. In GP–GC dyads, joint activities and face-to-face communication are essential to develop and maintain positive relationships [28, 29]. According to research from the USA, a typical face-to-face communication between young adult grandchildren and their grandparents lasts about 30 min and addresses topics such as family issues, grandchildren’s education, friends and leisure activities, as well as current world events [30]. Most grandchildren report being satisfied with the communication topics addressed with their grandparents [29, 30]. A survey among *N* = 104 grandparents showed that the older generation especially values joint activities with their grandchildren, with cooking, shopping and playing board and outdoor games being most gratifying for them [31]. Overall, joint leisure activities contribute to strengthening family ties and increase the quality of the GP–GC relationship [32].

Regarding communication frequency, research shows that face-to-face communication between grandparents and grandchildren typically occurs only about six times a year [33]. Communication frequency is higher if grandchildren live in geographical proximity of their grandparents, however, as grandchildren get older and more independent from family bonds it declines [34–36]. The inability of parents to facilitate direct contact between grandparents and young adult grandchildren, as well as grandchildren’s enrolment in study programs or employment, also contributes to the low frequency of face-to-face interactions between grandparents and young adult grandchildren [33, 37].

### Technology-mediated communication in grandparent–grandchild relationships

In addition to face-to-face communication, non-digital established media such as letters/postcards and landline phones are used in GP–GC relationships. They have the advantage of wide distribution and ease of use [3, 7, 38].

Digital established media are also increasingly adopted by the older generation and can therefore be used in GP–GC relationships. Older adults are using smartphones instead of landline phones more and more often, both for calling and texting [38]. They also use various forms of Internet-based communication such as email, social media platforms, and video conferencing [39, 40]. The successful use of digital media helps generate new conversational topics, reinforces family bonds, and promotes positive interaction among family members from different generations [41]. However, it requires both purchasing appropriate technical equipment as well as acquiring specific technology skills, both factors can be barriers for older adults [42, 43]. Nevertheless, the desire to keep in touch with grandchildren and stay involved in their lives is one of the main motivators for grandparents to overcome these barriers and go online [44–46].

Last but not least, there is an increase in practice and research projects that introduce innovative media such as AR- and social robot-based systems for older adults [18, 47]. These innovative media promise particularly rich communication and can thus benefit the GP–GC relationships. However, these technologies are not yet on the consumer market, which is why there is limited data on their use and impact in the context of GP–GC relationships.

## The present study

The spectrum of established media is changing very rapidly, especially in the area of digital media [48]. Current data on the variety of non-digital and digital media used in the GP–GC relationship mostly come from the USA, while findings from Germany are lacking. Hence, the present exploratory study focuses on media use in intergenerational communication in Germany. The first research question investigates the frequency of seven established communication forms (face-to-face, video conferencing, telephone, texting, social media, email, letter/postcard) in the GP–GC relationship from the perspective of young adult grandchildren:

- RQ1: How widespread is the use of different non-digital and digital media in GP–GC relationships?

Not only the very occurrence of direct or mediated contact is beneficial for GP–GC relationships, but also the perceived quality of communication. According to *Media Richness Theory*, media vary in their capability of efficient information transfer [49]. What determines the level of the respective medium’s richness is its ability to convey multiple communication cues, provide immediacy of feedback, language variety and personal focus [50]. Moreover, media differ in how much interpersonal closeness or *social presence* is experienced during mediated contact [51]. Media with a higher degree of social presence are usually being perceived as warm, personal, sensitive, sociable, and create an illusion of non-mediation [52]. Thereby, purely text-based and asynchronous communication (e.g., letters, emails) has the lowest media richness and social presence. Accordingly, communication that takes place synchronously and on an audio-visual level, such as video conferencing, conveys the highest media richness and social presence.

The question is, however, whether the theoretically predicted ranking of media is reflected in the subjectively experienced quality of communication between grandparents and grandchildren.

It should also be noted that the development of computer-mediated communication brought challenges and criticism to the theoretical concepts of media richness and social presence. In particular, *Social Information Processing Theory* suggests that technically lean media (e.g., letters or emails) can also generate a high degree of social presence if they are used competently and with suitable, empathetic choice of words to create interpersonal closeness [53]. Conversely, technically rich media can convey less social presence and eventually lead to a lower perceived communication quality if there are operating problems during use [54]. For instance, periodic image freeze during a video call could result in frustration and fatigue of participants and therefore, lead to less satisfactory communication experiences [55]. This brings up the subsequent research question:

- RQ2: How is the quality of communication via different non-digital and digital established media perceived in GP–GC relationships?

Innovative technologies can further improve the quality of communication in GP–GC relationships. Of particular interest are AR-based and social robot-based communication systems: Compared to existing digital media, they suggest a richer information exchange and a higher degree of social presence by conveying both audio and video cues, as well as the possibility to physically interact with the environment [57]. The perceived feeling of non-mediation is expected to foster social connectedness during the conversation and therefore, help maintain GP–GC relationships, while the possibility to interact with the environment allows for physical help and assistance which can be especially relevant for the older population.

While the landscape of innovative technology is wide, the focus of the present study on AR-based and social robot-based communication is motivated by their growing popularity in the context of use by and with older adults. Indeed, more and more AR-based and social robot-based systems have been recently developed for this age group, and multiple benefits for the physical and mental health of senior citizens are assumed and explored [14–18]. However, empirical proof of these benefits is often lacking. To address this gap, the present study investigates prospective AR-based and social robot-based communication, and their implications for GP–GC relationships:

- RQ3: How is the quality of communication via innovative media, i.e., AR-based and social robot-based systems, perceived in GP–GC relationships?

## Methods

The study entailed a cross-sectional online survey part and a randomized online experiment part, both conducted with young adult grandchildren as participants. In the survey part, grandchildren reported on the status quo of the non-mediated and mediated communication with their grandparents. In the experimental part, grandchildren evaluated vignettes of future communication scenarios with innovative technologies. The study is pre-registered: https://osf.io/dbvc8. The instrument, data set, statistical analysis script, and supplementary Tables S1-S6 are available at https://osf.io/fnbsw/.

### Participants and procedure

Participants were recruited between January and February 2021 on the campus of a small-town university in Germany (Technische Universität Ilmenau). Students were invited to participate in the study by their respective lecturers during study courses. Additionally, a link to the online survey with a short description of the study was distributed via university mailing lists. Participants were also encouraged to forward the survey link to their fellow students.

Participants needed to be students and have at least one living grandparent to be eligible for inclusion. A total of 289 university students participated in the two-part online study (survey plus experiment). Three respondents had to be eliminated due to implausible response patterns (e.g., extremely short completion time). This resulted in a final convenience sample of *N* = 286 students (18–30 years old, *M*_age_ = 22.86, *SD*_age_ = 3.23, 57% female). Sociodemographic characteristics of participants are presented in Table 1.

**Table 1:** Sociodemographic Characteristics of Grandchildren (= Study Participants)

Characteristics	*n*	*%*
*Age*		
18–24	213	74
25–29	55	19
30 +	18	6
*Gender*		
Male	115	40
Female	163	57
Diverse	5	2
Unknown	3	1
*Number of living biological grandparents*		
1	64	22
2	93	33
3	88	31
4	41	14
General technology competence		3.41 (0.86)^a^

*N* = 286. Percentage values are rounded

^a^–*M* (*SD*). Scale range: 1 (low competence)–5 (high competence)

At the beginning of the online study, participants were asked to select one grandparent with whom they had the most recent contact and provide all following answers focusing on this selected grandparent. This focus on one particular grandparent (as opposed to questions about grandparents in general) is necessary for valid measurement as grandchildren have separate relationships with different grandparents. Selected grandparents were between 61 and 100 years old (*M*_age_ = 79.23, *SD*_age_ = 6.70, 77% female) and, hence, fall in the category of older adults or senior citizens. Characteristics of grandparents reported on by the study participants are summarized in Table 2.

**Table 2:** Sociodemographic Characteristics of Grandparents (= Selected Grandparents Reported on by Study Participants)

Characteristics	*n*	*%*
*Age*		
50–59	0	0
60–69	24	8
70–79	105	37
80–89	138	48
90 +	19	7
*Gender*		
Male	65	23
Female	220	77
Other	1	0
*Lineage*		
Patrilineage	118	41
Matrilineage	167	58
Other	1	0
*Geographical distance from grandchild*		
Shared household	12	4
Same town/city in Germany	39	14
Another town/city in Germany, < 2 h drive	77	27
Another town/city in Germany, 2–4 h drive	73	26
Another town/city in Germany, 4–6 h drive	44	15
Another town/city in Germany, > 6 h drive	17	6
Outside of Germany	24	8
*Overall contact frequency grandchild with grandparent*		
(Almost) never	2	1
Less than once a year	6	2
Once a year	4	1
A few times a year	73	26
A few times a month	145	51
A few times a week	43	15
(Almost) daily	13	5
General technology competence		2.09 (1.02)^a^

*N* = 286. Percentage values are rounded

^a^–*M* (*SD*). Scale range: 1 (low competence)–5 (high competence)

The majority (71%) of participants reported having regular contact with selected grandparents at least a few times per month. However, since the beginning of the COVID-19 pandemic, face-to-face contacts became less frequent, while the frequency of mediated contacts raised: 40% of participants reported having mediated contact with their selected grandparents (much) more often in comparison with pre-pandemic times (see Table 3).

**Table 3:** COVID-19 Pandemic-Related Contact Frequency Changes in Grandparent–Grandchild Relationships

	Face-to-face contacts		Mediated contacts	
	*n*	*%*	*n*	*%*
Much less often	80	28	5	2
Less often	115	40	10	3
Unchanged	78	27	156	55
More often	4	1	95	33
Much more often	9	3	20	7

*N* = 286. Percentage values are rounded

Overall, participants reported good relationships with their selected grandparents (*M* = 4.05, *SD* = 0.79; on a scale from 1 = lowest relationship quality to 5 = highest relationship quality). In about half of the cases (46%), grandchildren and grandparents were equally likely to initiate communication. Otherwise, the initiative typically came from the grandchildren (29%), from their grandparents (14%) or from other family members (11%). Information about basic sociodemographic and relationship characteristics of grandchildren and grandparents reported in this section are necessary to contextualize the presented results on use and perceived quality of established and innovative communication technologies in GP–GC relationships.

### Instrument and measures

The online study used a carefully pretested instrument that was divided in three blocks of items and contained both closed and open-ended questions. In the first block, participants’ sociodemographic and GP–GC relationship characteristics were collected. The second block focused on current communication frequency and perceived communication quality with established media in the GP–GC relationship. The third block was dedicated to the assessment of innovative communication media and entailed an online vignette experiment addressing future AR- and social robot-based communication in the GP–GC relationship.

#### Sociodemographic and relationship characteristics

*Sociodemographic characteristics of the grandchildren* (= study participants) included their *age* (18–30 + years old), *gender* (male/female/diverse/not specified) and *number of living biological grandparents* (1–4).

*General technology competence of the grandchildren* was measured using the Technology Acceptance subscale of the Technology Commitment scale [56]. The subscale consisted of four statements related to technology use (e.g., “I am always interested in using the latest technical devices”) that were rated on a scale from 1 = *completely disagree* to 5 = *completely agree*. Items were averaged to produce a total mean score, higher scores indicated higher levels of general technology competence. Cronbach's *α* of the subscale in the original study was 0.84, and the present study showed the same reliability which is considered strong [57].

*Sociodemographic characteristics of the grandparents* (= selected grandparents reported on by the participants) included their *age* (50–100 + years old), *gender* (male/female/other), and *lineage* (patrilineage/matrilineage/other). Additionally, participants reported on the *geographical distance* between them and the selected grandparent. The seven response options to the question “How far away from your current residence does this grandparent live?” ranged from “shared household” to “outside of Germany” and were mostly measured in drive hours.

*Overall contact frequency between grandchildren and grandparents* was measured with a single item that asked “How often do you generally have contact with this grandparent (in any form)?” Seven response options ranged from 1 = *(almost) never* to 7 = *(almost) daily*.

*General technology competence of grandparents* was assessed on the same scale as technology competence of grandchildren. However, each item included the additional answer option *“don’t know”* as some grandchildren might find it problematic to accurately assess their selected grandparent’s general technology competence. The reliability of the scale was strong, Cronbach’s *α* = 0.91 [57].

*COVID-19 pandemic-related contact frequency changes in GP–GC relationships* were measured by asking “How did the contact with your grandparent change during the Corona pandemic compared to the time before?” Changes in face-to-face and mediated communication were measured separately on a scale from 1 = *much less often* to 5 = *much more often*.

*Quality of the GP–GC relationship* was measured with nine items associated with grandchildren’s emotional closeness to the selected grandparent (e.g., “I feel very close to this grandparent”), relationship satisfaction (e.g., “I am very satisfied with the relationship with this grandparent”), mutual help (e.g., “I very often receive support and help from this grandparent”) and getting along (e.g., “I can talk with this grandparent very easily”). Each statement was answered on a rating scale from 1 = *completely disagree* to 5 = *completely agree*. Items regarding relational closeness and relationship satisfaction were derived from similar studies conducted by Holladay and Seipke [3], and Harwood [7]. Items related to mutual help and getting along were adopted from Hartshorne and Manaster [58], and Cherlin and Furstenberg [59]. Items with negative polarity were reverse-coded. The resulting scale was represented by the average value of the selected items and ranged from 1 to 5 with higher scores indicating better relational quality. The scale showed high reliability of Cronbach’s *α* = 0.92 [57].

*Contact initiation in GP–GC relationships* was measured with a single item that asked “When you have contact with this grandparent (in any form), who typically takes the initiative?” Participants selected, whether the initiative for contact tends to come from *them/from the grandparent/equally from both sides/from parents or other relatives*.

#### Current communication with established media

Questions regarding frequency and quality of GP–GC communication with established non-digital and digital media were focused on seven forms of communication: (1) face-to-face, (2) letters/postcards, (3) phone (both landline and mobile), (4) email, (5) texting (e.g., SMS, WhatsApp, Telegram), (6) video conferencing (e.g., Zoom, Skype), and (7) social media platforms (e.g., Facebook, Instagram, Twitter).

*Communication frequency* was rated based on Harwood [7] on a scale from 1 = *(almost) never* to 7 = *(almost) daily*. Participants who reported never using a particular form of communication were automatically redirected to the next one. Others were asked to evaluate the selected medium regarding perceived communication quality.

*Communication quality* of each medium was assessed by separate single-item measures of *social presence* (“I feel very close to the grandparent during [this form of communication]”), *communication satisfaction* (“I am very satisfied with [this form of communication]”), and *perceived specific technology competence of grandparent* (“My grandparent manages [this form of communication] very well”). Each item was measured on a scale from 1 = *completely disagree* to 5 = *completely agree*, higher scores indicated higher communication quality.

#### Future communication with innovative media

The second part of the study contained a 2 (medium) × 2 (situation) between-subject online vignette experiment. Participants were randomly assigned to one of the four different hypothetical scenarios described in written form (see Table 4). The two innovative media (independent variable 1) included in the experiment were AR-based and social robot-based communication forms. The two situations included in the experiment (independent variable 2) were related to instrumental communication (the grandchild is helping the grandparent to set up a new vacuum cleaner) and socio-emotional communication (the grandchild is browsing an old family photo album together with the grandparent). Communication scenarios were developed during several discussion rounds with experts in the fields of communication, AR, cognitive robotics, electronic media, and audiovisual technology. The hypothetical situations were meant to be innovative and still realistic and easily comprehensible. The final version of all four scenarios was pretested to ensure comprehensibility, and the wording of the descriptions was improved based on the feedback of pretest participants.

**Table 4:** Design and Stimulus Material of the 2 (Medium) × 2 (Situation) Vignette Experiment

	AR-based innovative medium	Social robot-based innovative medium
Instrumental situation	Imagine that your grandparent has just bought a new vacuum cleaner, but can't manage it and asks you for help. A personal visit is not possible at the moment. You have to resort to media communication. As part of a project at Technische Universität Ilmenau, a new type of augmented reality-based communication system is available to you and your grandparent. This augmented reality system enriches the real environment acoustically and visually with virtual objects and persons and projects them as holograms onto a free space in the room. With the help of AR glasses, you and your grandparent can see and hear each other as if you were actually at each other's homes. The technical system is set up in such a way that you are virtually transported to your grandparent's home from their point of view, while you experience your grandparent at home. You can stand next to your virtual grandparent, take a close look at the vacuum cleaner, which is also virtual, and demonstrate the correct hand movements with your own hands. In this way, you can help your grandparent to start up the vacuum cleaner and insert the dust bin. For your grandparent, it is as if you were visiting and helping on the spot, embodied as a hologram.	Imagine that your grandparent has just bought a new vacuum cleaner, but can't manage it and asks you for help. A personal visit is not possible at the moment. You have to resort to media communication. As part of a project at Technische Universität Ilmenau, a new type of robot-assisted communication system is available to you and your grandparent. This system consists of a mobile robot with a robot arm, a camera and a screen. The robot is located in your grandparent's home and can be controlled remotely by you during the contact. Your grandparent can see and hear you through the robot's screen. By controlling the robot around the room, you can see and hear your grandparent and look around the room. With the robot, you can stand next to your grandparent, look closely at the vacuum cleaner and point to the right places on the vacuum cleaner with the robot arm that you can control remotely. This way you can help your grandparent to start up the vacuum cleaner and insert the dust bin. For your grandparent, it is as if you, represented by the robot, are visiting and helping on the spot.
Socio-emotional situation	Imagine that your grandparent has just found an old family photo album. They are very excited about it and want to look at it with you right away. A personal visit is currently not possible. You have to resort to media communication. As part of a project at Technische Universität Ilmenau, a new type of augmented reality-based communication system is available to you and your grandparent. This augmented reality system enriches the real environment acoustically and visually with virtual objects and persons and projects them as holograms onto a free space in the room. With the help of AR glasses, you and your grandparent can see and hear each other as if you were actually at each other's homes. The technical system is set up in such a way that you are virtually transported to your grandparent's home from their point of view, while you experience your grandparent at home. You can sit next to your virtual grandparent on the sofa, look closely at the virtual photo album and point with your finger at objects or people in the photos that you are talking about with your grandparent. This way you can keep your grandparent company while looking at the photo album. For your grandparent, it is as if you are visiting them as a hologram and keeping them company.	Imagine that your grandparent has just found an old family photo album. They are very excited about it and want to look at it with you right away. A personal visit is currently not possible. You have to resort to media communication. As part of a project at Technische Universität Ilmenau, a new type of robot-assisted communication system is available to you and your grandparent. This system consists of a mobile robot with a robot arm, a camera and a screen. The robot is located in your grandparent's home and can be controlled remotely by you during the contact. Your grandparent can see and hear you through the robot's screen. By controlling the robot around the room, you can see and hear your grandparent and look around the room. With the robot, you can stand next to your grandparent sitting on the sofa and use the robot arm, which you can control remotely, to point at objects or people in the photos that you are talking about with your grandparent. This allows you to keep your grandparent company while looking at the photo album. For your grandparent, it is as if you, represented by the robot, are visiting and keeping them company.

After getting familiar with the assigned scenario, participants were asked to evaluate it in terms of *social presence* (“I would feel very close to the grandparent during this form of communication”), *communication satisfaction* (“I would be very satisfied with this form of communication with the grandparent”) and *perceived specific technology competence of their grandparent* (“My grandparent would manage this form of communication very well”). Each item was rated on a scale from 1 = *completely disagree* to 5 = *completely agree*. Higher scores indicated higher degrees of communication quality. Additionally, two open-ended questions asked generally about the perceived advantages and disadvantages of the AR-based and social robot-based innovative media.

### Data analysis

Data were analyzed using descriptive and inferential statistics. All statistical analyses were performed in R version 4.1.2. Significance level was set at 5%, and Bonferroni correction was applied for multiple testing.

First, means and standard deviations were calculated to assess the average frequency of use of each of the seven communication forms (RQ1). Although the frequency scale from 1 = *(almost) never* to 7 = *(almost) daily* technically is ordinal, in line with previous research from the field (e.g., [3, 7, 38]), it was treated as an interval scale, thus justifying the use of mean values as measures of central tendency [60]. Differences in average communication frequency of established media were then compared using one-way repeated measures analysis of variance (ANOVA) with forms of communication as a within-subjects factor. Social media and email responses showed very low variability within the variables; therefore, the decision was made to exclude them from the inferential statistical analysis, as such extreme deviations from normality could affect the validity of the inferential statistic [61]. Remaining data did not show major deviations from normality, based on visual inspection of quantile–quantile (Q–Q) plots. There were no extreme outliers in the data. Mauchly’s test indicated that the assumption of sphericity of the data was violated (*W* = 0.67, *p* < 0.001, *ε* = 0.83); therefore, degrees of freedom were adjusted using Greenhouse–Geisser estimates of sphericity.

One-way multivariate repeated measures ANOVA (MANOVA) was performed to answer RQ2 with forms of communication as predictor variables and three communication quality measures (communication satisfaction, social presence and perceived specific technology competence of grandparent) as outcome variables. Due to the filtering of the questionnaire, social media and email communication forms received very low numbers of observations and therefore, were not included in further analyses. Six multivariate outliers were identified using Mahalanobis distance as a criterion and were excluded. Multivariate normality was examined graphically by firstly checking Q–Q plots for each dependent variable separately, and then by examining the Q–Q plot of the standardized residuals. The assumption of multivariate normality was satisfied.

Two-way factorial MANOVA was conducted to compare the differences in evaluations of virtual scenarios (RQ3). Two independent factor variables were medium (AR or social robot) and situation (vacuum cleaner or photo album). Communication quality measures (communication satisfaction, social presence and perceived specific technology competence of grandparent) were dependent variables. Four multivariate outliers were identified using Mahalanobis distance as a criterion and were excluded. Multivariate normality was examined graphically by firstly checking Q–Q plots for each dependent variable separately, and then by examining the Q–Q plot of the standardized residuals. The assumption of multivariate normality was met. Homogeneity of covariance matrices was assessed visually by plotting the standardized residual versus fitted responses, and then by performing Levene’s test for homogeneity of variance for each dependent variable separately. All tests confirmed that data were homogeneous.

Responses to open-ended questions regarding perceived advantages and disadvantages of innovative media were analyzed qualitatively using an inductive *Thematic Analysis* approach [62]. This approach was selected as it allows for a theoretically flexible data-driven analysis. Firstly, short codes related to each response were created. Then, the codes were examined for common themes. To ensure reliability, 15% of all the responses were randomly selected and coded by two independent coders according to identified themes. All themes showed inter-coder agreements of 93% and above. Cohen’s kappa coefficients varied between 0.76 and 1 which indicates substantial to almost-perfect agreement [63].

Ultimately, all responses were coded into themes. In cases where a response mentioned multiple advantages or disadvantages, multiple themes were coded. Answers that did not mention any advantages or disadvantages or those that were unclear were excluded from the analysis. Additionally, frequencies of all themes were calculated.

## Results

### Communication frequency with established media

RQ1 asked about communication frequency with established media. Overall, face-to-face and phone conversations occurred most frequently in GP–GC relationships, with about 85% of participants engaging in them at least a few times per year. The least frequently used established media were social media and email—more than 90% of all surveyed grandchildren reported not using them at all to communicate with the selected grandparents.

Average frequencies of each communication form are presented in Table 5. One-way repeated measures ANOVA showed significant differences between the communication forms, *F*(3.31,944.67) = 210.11, *p* < 0.001, *η*^*2*^*G* = 0.34. Post hoc pairwise comparisons revealed that face-to-face and phone communication were both used significantly more often than texting, video conferencing and letters/postcards.

**Table 5:** Communication Frequency with Established Non-digital and Digital Media

Communication form	Total (*N* = 286)		< 2 h drive (*n* = 128)		> 2 h drive (*n* = 158)	
	M	SD	M	SD	M	SD
Telephone	4.24_a_	1.29	4.34_c_	1.19	4.15_a_	1.36
Face-to-face	4.19_a_	1.17	4.76_a_	1.01	3.73_b_	1.08
Texting	2.77_c_	1.98	2.89_d_	2.03	2.67_c_	1.93
Video conferencing	1.98_b_	1.52	1.67_b_	1.34	2.22_ cd_	1.62
Letters/postcards	1.85_b_	1.05	1.79_b_	1.02	1.90_d_	1.07
Email	1.26	0.84	1.19	0.68	1.32	0.96
Social Media	1.13	0.68	1.13	0.71	1.12	0.66
	*F*(3.31,944.67) = 210.11, *p* < .001, *η*^*2*^*G* = .34		*F*(3,380.84) = 156.24, *p* < .001, *η*^*2*^*G* = .47		*F*(3.28,515.03) = 81.38, *p* < .001, *η*^*2*^*G* = .26	

Communication frequency was measured on a Likert scale ranging from 1 = (almost) never to 7 = (almost) daily. Means with different subscripts within the same column are significantly different (*p* < .01 based on pairwise comparisons with Bonferroni correction for multiple testing). Means without subscripts were excluded from inferential statistical analysis due to extreme low variability of observations

In order to validate the ranking, additional comparisons were made based on the geographical distance between grandchildren and grandparents. Results indicate that participants who live less than a two-hour drive away from their grandparents engage in face-to-face communication significantly more often than in communication over the phone. Those who live further away, engage in phone conversations significantly more often than in face-to-face communication.

General technology competence and gender of both grandparents and grandchildren did not show any considerable effect on the resulting frequency ranking (see supplementary Tables S1 and S2 at https://osf.io/fnbsw/).

### Communication quality with established media

RQ2 asked about communication quality related to established media. Results show that face-to-face and phone communication in the GP–GC relationship were associated with higher communication satisfaction and a higher degree of social presence compared to other communication forms (see Table 6).

**Table 6:** Communication Quality Related to Established Non-digital and Digital Media

Communication form	*n*	Communication satisfaction		Social presence		Perceived specific technology competence of grandparent	
		*M*	*SD*	*M*	*SD*	*M*	*SD*
Face-to-face	271	4.07_a_	0.93	3.98_a_	0.94	4.36_a_	0.80
Telephone	262	3.75_b_	0.92	3.30_b_	0.96	4.12_b_	0.89
Letters/postcards	135	3.51_c_	1.09	2.75_c_	1.19	4.38_a_	0.94
Texting	138	3.40_c_	1.08	2.65_c_	1.00	3.56_c_	1.18
Video conferencing	97	3.36_c_	1.12	3.18_b_	1.03	3.05_d_	1.06
Email	28	3.21	1.23	2.18	1.02	3.79	1.10
Social Media	11	3.18	1.08	3.00	1.10	3.36	1.12
		*F*(4,611) = 29.37, *p* < .001, *η*_*p*_^*2*^ = .16		*F*(4,611) = 97.62, *p* < .001, *η*_*p*_^*2*^ = .39		*F*(4,611) = 67.02, *p* < .001, *η*_*p*_^*2*^ = .30	

Communication quality was measured on a Likert scale ranging from 1 = low quality to 5 = high quality. Means with different subscripts within the same column are significantly different (*p* < .01 based on pairwise comparisons with Bonferroni correction for multiple testing). Means without subscripts were excluded from inferential statistical analysis due to low number of observations

A one-way repeated measures MANOVA confirmed significant differences in the assessment of all three communication quality measures, *F*(12,1833) = 42.70, *p* < 0.001, *η*^*2*^ = 0.65. A series of one-way repeated measures ANOVAs on each of the three dependent variables was conducted as follow-up tests to the MANOVA. As seen in Table 6, all tests were significant, demonstrating large effect sizes [64]. Post hoc pairwise comparisons with Bonferroni correction for multiple testing were performed for each measure of communication quality. Results revealed that respondents evaluated face-to-face communication significantly better than the other mediated forms of communication in terms of all three communication quality measures.

To control for possible gender effects, additional analyses were run separately for grandparents and grandchildren. Although grandfathers were ascribed slightly higher general technology competence levels than grandmothers, it did not affect the overall quality ranking of the communication forms. No gender effects were observed between male and female grandchildren as well (see supplementary Tables S3 and S4 at https://osf.io/fnbsw/).

### Communication quality related to innovative media

#### Results of quantitative analysis

RQ3 asked about communication quality related to innovative media in a randomized online vignette experiment. Descriptive results for each scenario are summarized in Table 7. Again, separate analyses were run for grandparents and grandchildren to control for possible gender effects. Grandfathers were ascribed slightly higher technology competence than grandmothers. However, these differences did not have an effect on the overall evaluations of scenarios. Gender of grandchildren also did not affect the results (see supplementary Tables S5 and S6 at https://osf.io/fnbsw/).

**Table 7:** Communication Quality Related to Innovative Media

Medium	Situation	*n*	Communication satisfaction		Social presence		Perceived specific technology competence of grandparent	
			*M*	*SD*	*M*	*SD*	*M*	*SD*
AR	Vacuum cleaner	74	3.15	1.17	3.11	1.23	1.95	1.06
	Photo album	81	3.16	1.10	2.87	1.21	2.00	1.06
Social Robot	Vacuum cleaner	62	2.95	1.18	3.21	1.10	2.52	1.08
	Photo album	69	2.49	1.02	2.51	0.87	2.03	1.00
Total		286	2.95	1.14	2.94	1.14	2.10	1.07

Communication quality was measured on a Likert scale ranging from 1 = low quality to 5 = high quality

Differences in participants’ communication quality assessments of the scenarios were examined using a 2 (medium) × 2 (situation) factorial MANOVA. One significant main effect for the factor medium (AR-based versus social robot-based) emerged, *F*(3,276) = 14.77, *p* < 0.001, *η*^*2*^ = 0.14. There was no significant effect for the factor situation (instrumental: vacuum cleaner versus socio-emotional: photo album), *F*(3,276) = 1.90, *p* = 0.13, *η*^*2*^ = 0.02, and no interaction effect, *F*(3,276) = 1.60, *p* = 0.19, *η*^*2*^ = 0.02.

A series of one-way ANOVAs was conducted for each of the communication quality measures as a follow-up to the significant results. All conducted tests were significant and showed that the AR scenarios were evaluated significantly better than the social robot scenarios in terms of communication satisfaction and social presence. However, the perceived specific technology competence of grandparents was significantly higher for the robot-based as opposed to the AR-based scenarios (see Table 8).

**Table 8:** Communication Quality of Augmented Reality-Based vs. Robot-Based Systems

Communication quality measure	AR (*n* = 153)		Social Robot (*n* = 129)		*F*(1,278)	*η*^*2*^*G*
	*M*	*SD*	*M*	*SD*		
Communication satisfaction	3.18	1.11	2.71	1.11	11.77^**^	.04
Social presence	3.17	1.16	2.67	1.04	13.67^**^	.05
Perceived specific technology competence of grandparent	1.96	1.03	2.25	1.04	6.11^*^	.02

Communication quality was measured on a Likert scale ranging from 1 = low quality to 5 = high quality. **p < .001, *p < .05

#### Results of qualitative analysis

##### Advantages

When evaluating the four different communication scenarios based on open-ended questions, participants mentioned seven main advantages of AR- and social robot-based innovative media in the GP–GC relationship: (1) higher degree of social presence, (2) improved communication quality, (3) possibility of physical interaction, (4) convenience of use, (5) more frequent communication, (6) health protection, and (7) joy of use. All seven mentioned advantages, illustrative examples, and frequency of coding are summarized in Table 9, separately for AR- and social robot-based scenarios.

**Table 9:** Main Advantages of Innovative Media in Grandparent–Grandchild Relationships

Theme	Description	Example	AR %	Social robot %
(1) Higher degree of social presence	Feeling of being in the same room with the grandparent, closeness to grandparent	“You don't have to be there, but you still have the feeling of being close to the grandparents because you physically see each other” “(possibly) more the feeling of actually being there”	25	7
(2) Improved communication quality	Better quality of communication, both technical (audio and video quality) and emotional (more personal feeling)	“Gestures and facial expressions can be conveyed better, sound perception is possibly better” “Feels more personal than other options”	25	3
(3) Possibility of physical interaction	Practicality of innovative media: possibility to physically interact with environment and provide help and assistance for grandparent	“You can point to something. This sometimes helps in communication, you don't always have to describe what you mean” “The possibility to point to things and clarify explanations in this way”	14	28
(4) Convenience of use	Convenience and ease of use, both for grandchild and grandparent	“Since the robot can be controlled by me, my grandparents don't have to deal with the technology” “If I 'visit' my grandparent via AR instead of coming over in person, I save time, money, and pollute the environment less by skipping the actual trip”	5	8
(5) More frequent communication	Possible increase in frequency of communication with the grandparent	“We could have more frequent contact with each other, as would not be possible due to distance, and it would become more attractive to be in contact with my grandmother more often than is currently possible with phone calls” “You can see each other more often despite the distance (study)”	3	2
(6) Health protection	Using innovative media could help protect grandparent against infections	“The health could be less endangered from possible contagious diseases” “You do not put anyone in danger because of possible infection”	2	4
(7) Joy of use	A new exciting form of communication	“Exciting experience” “Controlling a robot is definitely fun”	2	2

*N* = 176 statements about perceived advantages. Themes are presented in descending order, according to the frequency of their mentioning in analyzed responses

As illustrated in Table 9, participants who were assigned to AR scenarios named better social presence and overall communication quality as main advantages of this innovative medium. In particular, participants mentioned that the AR headset could help them communicate with their grandparents more effectively than using established media (e.g., “*This allows you to communicate better through body language, gestures or with facial expressions, for example, unlike a phone call”).* Social robot-based scenarios were more frequently complimented for the possibility to physically interact with the environment (e.g., *“You can point to something. This sometimes helps in communication, you don't always have to describe what you mean”*).

Another advantage of communication scenarios involving a social robot was seen in the convenience of its use. Although quantitative results highlighted overall low perceived specific technology competence of grandparents in dealing with a robot, about 8% of participants' statements mentioned the fact that the social robot could be controlled remotely by a grandchild which would spare grandparents the need to learn how to operate this new technology (e.g., *“Since the robot can be controlled by me, my grandparents don't have to deal with the technology”*)*.*

##### Disadvantages

When evaluating the four different communication scenarios based on open-ended questions, participants mentioned six main disadvantages of AR- and social robot-based innovative media in the grandparent–grandchild relationship: (1) difficulty to use, (2) high cost, (3) artificial feeling, (4) negative health impact, (5) replacement of personal contact, and (6) uselessness (see Table 10).

**Table 10:** Main Disadvantages of Innovative Media in Grandparent–Grandchild Relationships

Theme	Description	Example	AR %	Social robot %
(1) Difficulty to use	Innovative media is difficult and overwhelming to use for grandparent and/or grandchild	“Difficult operation/use for grandparents if they are not too technically proficient (in the sense of using the settings, starting the communication, …)” “My grandparents would probably get frustrated quickly and throw the robot out the window”	44	32
(2) High cost	Device is expensive to purchase and/or use	“Requires grandparents to have an Internet connection. Purchase is definitely more cost-intensive than telephone or equipment for video conferencing” “Is it worth the cost?"	7	7
(3) Artificial feeling	Communication is perceived as unnatural and cold	“I would be there and not there at the same time, it would not feel good” “Sounds somehow more impersonal and alienating than regular videoconferencing”	5	11
(4) Negative health impact	Using innovative media could have negative effect on physical and/or mental health of grandparent	“Body might react badly to it (headache, dizziness)” “Possible sadness of grandparents that you are not really there in person”	4	3
(5) Replacement of personal contact	Personal visits will become less frequent or completely replaced by media	“This would perhaps limit the personal contact even more because it would be more practical to use, no travel time and costs, etc., and I find it sad” “Carries the risk of reducing face-to-face (on-site) communication (for the grandchild's convenience)”	3	1
(6) Uselessness	Innovative media is meaningless, won’t provide benefits compared to existing and will be perceived as useless by grandparent	“One more device that doesn’t work” “It could be that my grandfather sees a robot as a technical gadget that is not useful for anything. And that it would confuse my grandmother too much if a robot with my face on it drove around the apartment”	1	6

*N* = 189 statements about perceived disadvantages. Themes are presented in descending order, according to the frequency of their mentioning in analyzed responses

As illustrated in Table 10, perceived disadvantages did not differ a lot between the two innovative technologies: Both for AR- and social robot-based scenarios participants named difficulty to use and overall low acceptance as main issues. Some participants were doubtful that their grandparents would agree to try the device at all (e.g., *“My grandma would most likely refuse to try it and (if she did) would have a very hard time with it. She has no experience with new technologies and no interest in them”*). Moreover, some participants (about 5% for the AR- and 11% for the social robot-based scenarios) stated that such forms of communication would feel cold and artificial in comparison with face-to-face contacts (e.g., *“It is not the same”*).

In addition, many grandchildren focused on the practical side of having an AR- or social robot-based communication device at home. About 7% of the respondents' statements stressed the importance of considering additional expenses involved, both in terms of purchasing the device itself and operating costs (e.g., *“In her apartment, [my grandmother] doesn't have Wi-Fi or a cell network. Everything would have to be set up, contracts signed and then she would have to pay extra costs”*).

## Discussion

The aim of the study was to investigate the frequency (RQ1) and quality (RQ2) of GP–GC communication using different non-digital and digital established media, as well as to explore the potential use of innovative media (RQ3) from the perspective of young adult grandchildren. An online survey and a randomized online experiment were conducted among a sample of university students in Germany. Grandchildren reported having very positive relationships with their selected grandparents and maintained regular contact with them at least a few times a month.

Face-to-face visits and phone calls were the most frequently used forms of communication in GP–GC relationships. Face-to-face communication was more frequent among participants who live within a two-hour drive distance from the selected grandparent, while phone calls were more common among those who live further away. Such findings are in line with earlier studies conducted in the USA [3, 7, 38]. However, they also show that the use of texting has largely increased in recent years, while the use of email has decreased. Although not based on representative data, these results contribute to previous studies that show that older adults are becoming more and more technologically competent and eager to use modern digital media to stay in touch with their grandchildren (e.g., [44]).

Regarding perceived communication quality, face-to-face and phone communication were evaluated best. As Media Richness Theory suggests, non-mediated contacts should have the highest quality of communication and the highest degree of social presence; therefore, the high ranking of face-to-face communication was expected. The high ranking of phone communication, on the other hand, is not explained by Media Richness Theory. Lack of visual cues during a telephone conversation should lead to a lower degree of social presence, which, in turn, is expected to lower the overall communication satisfaction. It is, however, important to keep in mind that the telephone is a popular and widely available medium that has been used by older adults for decades. Social Information Processing Theory suggests that certain limitations of a leaner medium can be overcome if its being used with confidence and in appropriate social situations [65]. The level of personal closeness between communication partners can also influence the richness of information exchange. Therefore, grandparents’ previous experience with the medium combined with their positive relationship with grandchildren can foster satisfactory communication experiences with a lean media channel.

Technically richer forms of communication, such as video conferencing, received notably lower communication satisfaction ratings. This result was unexpected in the context of Media Richness Theory because synchronous audio-visual communication is expected to provide rich communication experiences. However, regardless of the fairly high degree of social presence, participants reported quite low satisfaction with video conferencing. One possible explanation can be that grandparents’ lack of technology competence and experience with this medium makes communication via video conference apps too complicated and uncomfortable for them. The uneasy feeling does not allow for a smooth communication flow, eventually lowering communication satisfaction for both grandparents and grandchildren. Observed low perceived specific technology competence of grandparents supports this explanation, as do the assumptions of Social Information Processing Theory. The effectiveness and quality of mediated communication apparently rely on the user’s personal experience with a certain medium.

Innovative digital media, namely AR- and social robot-based systems, were presented to participants in an online vignette experiment describing two different scenarios, in which each medium was used. Both, AR and social robots, allow communication partners to see and hear each other, interact nonverbally and interact with the environment together (e.g., to point on objects or move together within the room). According to Media Richness Theory, the affordances of innovative media imply a high level of richness and social presence. However, all innovative media scenarios received lower social presence scores compared to established media. Similar to the communication quality with established digital media (e.g., video conferencing), these lower evaluations were related to low perceived specific technology competence of grandparents. Participating grandchildren had strong reservations regarding their grandparents’ ability to operate innovative media. At the same time, they did not dismiss the benefits of AR- and social robot-based communication and named a lot of advantages, which allows for a positive prognosis for the future AR- and social robot-based communication in GP–GC relationships.

## Limitations and outlook

The study offers valuable insights into the current status of GP–GC relationship in Germany; however, it is not without limitations. Firstly, the convenience sampling procedure limits generalizability of the results. Moreover, university students as study participants could affect study results in the sense that they might be more familiar with modern technologies and have an overall more positive attitude toward them compared to younger adults with different educational or economic backgrounds. The results of this study should therefore be regarded with caution and the findings should be considered preliminary. A follow-up study with a representative sample of younger adults is recommended to confirm the findings.

Furthermore, the online experiment was based on written vignettes, which could have potentially led to misunderstandings that affect the evaluation of the hypothetical scenarios. While written vignettes are suitable and common for online experiments, future research should use video- or prototype-based demonstrations of the scenarios. An investigation of digital media use in GP–GC relationships from the perspective of grandparents will also provide interesting insights and fruitful topics for discussion.

## Conclusion 

Frequent and positive communication is an integral part of intergenerational relationships [3]. Established and innovative communication media can support this communication in situations when personal visits become problematic. Largely based on the assumptions of *Media Richness Theory*, the present study demonstrates its limitations in the context of GP–GC relationships. Despite technically rich information exchange, modern digital media, such as video conferencing, were not able to provide satisfactory communication experiences between grandparents and grandchildren. At the same time, the telephone as a lean medium seemed to sufficiently fulfill communication needs of both parties. Based on the findings, it seems fruitful to integrate technology-focused and human-focused theories to predict successful and satisfactory media use in intergenerational communication. Most likely, meaningful and high-quality technology-mediated communication between grandparents and grandchildren will occur not only when a rich media channel is used, but also when the respective media channel is used with confidence. The observed perceived low technology competence of grandparents supports this notion, as it was the main factor that negatively affected satisfaction with modern digital forms of communication.

Moreover, participants’ concerns regarding AR-based and social robot-based communication being artificial, cold and eventually leading to the grandparent feeling lonelier than before should not be disregarded. The future development of innovative communication technologies for intergenerational use should therefore follow a human-centered design process that acknowledges the needs and fears of both grandparents and grandchildren. Particular attention should be paid to utility, usability and joy of use of innovative technologies that are to be used by the older generation.

## Data Availability

The instrument, data set, statistical analysis script as well as supplementary tables S1-S6 are available in the OSF repository, https://osf.io/fnbsw/.
